# Is reappraisal always effective in modifying emotional reactions in females? The role of regulatory timing and goals

**DOI:** 10.1002/brb3.911

**Published:** 2018-01-11

**Authors:** Chunping Yan, Na Lin, Lixia Cui, Qin Zhang

**Affiliations:** ^1^ Department of Psychology Learning and Cognition Key Laboratory of Beijing Capital Normal University Beijing China; ^2^ Department of Psychology Xinxiang Medical University Xinxiang China

**Keywords:** cognitive reappraisal, emotion regulation, emotion regulatory goal, event‐related potential, regulatory timing

## Abstract

**Introduction:**

Numerous studies have explored the effect of cognitive reappraisal before or after emotion‐inducing events. However, only a few studies have examined the influence of regulatory timing on the effectiveness of reappraisal. Thus, the aim of this study was to investigate the role of regulatory timing and goals in reappraisal regulation, which would help promote the specific application of cognitive reappraisal in emotion regulation. We hypothesized that decrease reappraisal would be more effective when initiated early rather than late, but increase reappraisal would be more effective when initiated in the emotional high‐activation phase.

**Methods:**

This study, via event‐related potential (ERP) technique, probed the influence of the timing and regulatory goal on negative emotion when reappraisal was introduced, respectively 500 ms before (anticipatory), 2,000 ms after (online 2,000 ms) picture onset (in Experiment 1), 500 ms after (online 500 ms) picture onset, and 1,500 ms after (online 1,500 ms) picture onset (in Experiment 2).

**Results:**

Based on the ERP results, under the anticipatory regulation condition, the LPP amplitude in the parietal area was significantly reduced by decrease reappraisal during 700–2,100 ms after picture onset, and under the online 500 ms regulation condition, the LPP in central and parietal areas was significantly enhanced by increase reappraisal during 450–750 ms after regulatory cue onset. Moreover, our results showed that increase reappraisal evoked a larger prefrontal or frontal LPP than decrease reappraisal beginning at about 700 ms after picture onset under the anticipatory regulation condition and beginning at 450 ms after regulatory cue onset under the online 500 ms regulation condition, which may reflect increased cognitive effort and mental conflict associated with increase reappraisal.

**Conclusion:**

The anticipatory reappraisal successfully decreased negative emotion, and online 500 ms reappraisal successfully increased negative emotion. Our results support the hypothesis.

## 1 INTRODUCTION

Emotion regulation plays an important role in maintaining one's well‐being and appropriate functioning (Gross, 1998b). For this reason, the effectiveness of various emotion regulation strategies has been the focus of numerous studies. Cognitive reappraisal, one of the most flexible and effective regulation strategies, entails cognitive attempts at changing the meaning of emotion‐inducing events (Gross, 1998a; Gross & Thompson, 2007). Previous studies have shown that by modifying the interpretation of emotion‐inducing events, cognitive reappraisal can change (increase or decrease) the individual's subjective emotional experience (Kalisch et al., 2005; McRae, Ciesielski, & Gross, 2012; Ochsner, Bunge, Gross, & Gabrieli, 2002; Ochsner et al., 2004; Ray, McRae, Ochsner, & Gross, 2010; Wager, Davidson, Hughes, Lindquist, & Ochsner, 2008; Wessing et al., 2015), facial muscle activity (Deveney & Pizzagalli, 2008; Jackson, Malmstadt, Larson, & Davidson, 2000; Ray et al., 2010), and physiological responses (McRae et al., 2012; Parvaz, Moeller, Goldstein, & Proudfit, 2014; Wessing et al., 2015). However, is initiating reappraisal before and during the emotion generative process equally effective in changing emotional responses?

According to Gross's (1998b) process model of emotion regulation, cognitive reappraisal is categorized as an antecedent‐focused strategy which starts operating before the response tendencies have been fully activated. This implies that reappraisal should be more effective when the instruction to reappraise is delivered before rather than after the emotion‐inducing event, or when it delivered early rather than late during the emotion generative process, because of the generally lower level of emotional activation at that stage (Gross & Thompson, 2007; Urry, 2009). Sheppes and Meiran (2007) defined the attempt to change an emotion during an emotion‐inducing event as online emotion regulation and suggested that employing reappraisal late may be less effective in decreasing negative emotion because the high level of emotional activation makes it difficult to override the previously formed affect‐laden train of thought and establish a new train of thought that would reduce negative emotion. They also proposed that there is a “point of no return,” at which emotion is fully activated and therefore reappraisal may become exceptionally difficult.

Some of the previous studies on the subject presented the reappraisal instruction to participants before the emotion‐inducing event (e.g., film, picture) (Gross, 1998a; Kalisch et al., 2005; Kim & Hamann, 2007; Ochsner et al., 2004; Schönfelder, Kanske, Heissler, & Wessa, 2014), some delivered the reappraisal instruction during the emotion‐inducing event (Deveney & Pizzagalli, 2008; Eippert et al., 2007; Jackson et al., 2000; Ochsner et al., 2002; Sheppes, Catran, & Meiran, 2009; Urry et al., 2006; Van Reekum et al., 2007), and others delivered the instruction after the emotion‐inducing event (Ray, Wilhelm, & Gross, 2008; Rusting & Dehart, 2000). However, only a few studies examined the influence of regulatory timing on the effectiveness of reappraisal. The first such study, conducted by Sheppes and Meiran (2007), explored the online regulation effect of cognitive reappraisal by manipulating the strategy initiation point during sadness‐evoking films either in advance (10 s before the film started), early (37.5 s after the film started), or late (114.0 s or 190.0 s after the film started). The results showed that reappraisal was more effective in reducing the negative experience when initiated in advance or early rather than late. The authors argued that the lower success of late reappraisal may suggest a “point of no return.”

Urry (2009) further investigated whether emotion regulatory success is moderated by regulatory timing using negative pictures as emotion‐inducing stimuli. Cognitive reappraisal was introduced 2 s before (anticipatory timing) or 4 s after (online timing) picture onset. Contrary to the result of Sheppes and Meiran (2007), Urry found that decrease reappraisals produced weaker unpleasant experience relative to the maintenance condition, but this effect did not depend on the regulatory timing. Compared to the maintenance condition, increase reappraisals produced stronger unpleasant experience and increased corrugator muscle activity, heart rate, and electrodermal activity. And higher corrugator muscle activity and electrodermal activity elicited by increased reappraisals were only apparent under the online timing condition. These results supported the notion that the impact of regulatory timing depends on one's regulatory goal. Urry (2009) argued that the different effects of early and late reappraisal introduction are caused by different emotional activation levels, and when one intends to increase the emotional response, online regulation may be most effective way because the high emotional activation that is already present is consistent with the increase regulatory goal. However, one possible reason for the effect of decrease reappraisals being not influenced by the regulatory timing is that the effect of regulatory timing may be small, and self‐reported negative emotion is not sensitive enough to the small effect. In addition, Urry (2009) did not observe any effects of decrease reappraisals on expressive behavior or autonomic physiology, which was inconsistent with many previous studies (Deveney & Pizzagalli, 2008; Jackson et al., 2000; Kim & Hamann, 2012; McRae et al., 2012; Ray et al., 2010). Therefore, it is necessary to further examine the influence of regulatory timing on reappraisal effects by employing other measurements, as this study aimed to do.

In addition to regulatory timing, this study also manipulated the reappraisal goal, which included increasing negative emotion, decreasing negative emotion, and simply viewing negative pictures. Previous studies have confirmed the effects of regulatory goals through various measures (Moser, Hajcak, Bukay, & Simons, 2006; Moser, Krompinger, Dietz, & Simons, 2009; Ray et al., 2010; Urry, 2009; Webb, Gallo, Miles, Gollwitzer, & Sheeran, 2012; Wu, Tang, Huang, Hu, & Luo, 2013). For example, in Ray et al. (2010), participants were asked to view negative pictures under increase, decrease, or maintenance conditions of negative emotions, and their saccades, electrical responses of corrugator muscles, and subjective feelings were recorded. The results showed that the negative emotional experience was strongest under the increase condition and weakest under the decrease condition. The saccades and corrugator muscle activity were also significantly higher under the increase condition than under the decrease or maintenance condition, and the corrugator muscle activity was lowest under the decrease condition.

Because of the high temporal resolution of event‐related potentials (ERPs), this study used ERPs to provide an online measure of the reappraisal process to determine the best timing of increase or decrease reappraisal. ERPs have been used in some previous studies on reappraisal. ERPs response to unpleasant pictures can be modulated by cognitive reappraisal, and previous studies focused on the effects of reappraisal instructions on the amplitude of the late‐positive potential (LPP). LPP is a broad positive component sensitive to the motivational relevance of visual stimuli (Cuthbert, Schupp, Bradley, Birbaumer, & Lang, 2000; Schupp, Junghofer, Weike, & Hamm, 2004; Schupp et al., 2000). The previous studies (Cuthbert et al., 2000; Gao et al., 2017; Moser et al., 2009; Zhang et al., 2012, 2013) suggested that the central‐parietal and parietal LPP in general was associated with emotional significance of stimuli, the more positive parietal LPP indicating the enhancement in emotional intensity and the more negative parietal LPP indicating the decrease in emotional intensity. However, several recent studies found higher frontal LPP under reappraisal relative to the viewing condition and argued that enhanced frontal LPP may index increased cognitive effort associated with reappraisal (Bernat, Cadwallader, Seo, Vizueta, & Patrick, 2011; Moser, Hartwig, Moran, Jendrusina, & Kross, 2014; Shafir, Schwartz, Blechert, & Sheppes, 2015). Therefore, this study also mainly explored the effect of reappraisal on LPP amplitude at frontal, central, and parietal area.

Some studies have inspected the effects of emotion regulation by central and parietal LPP suggesting emotional intensity. For instance, Moser et al. (2006) examined the effects of emotion regulation (merely viewing or decreasing/enhancing emotional responses to negative pictures) using ERPs. They found that compared to the enhancement and passive viewing conditions, LPP was smaller in the decrease condition, and the effect lasted for several hundred milliseconds from 250 ms after the stimulus onset. Hajcak and Nieuwenhuis (2006) asked participants to reinterpret unpleasant pictures to decrease their negative responses. Their results also showed decreased LPP amplitude from 200 ms to 1,800 ms after picture onset in the reappraisal condition, with no such decrease in the viewing condition, and the degree of LPP reduction was positively related to reductions in self‐reported emotional intensity that followed the reappraisal instruction. Moser et al. (2009) examined the ERP correlates of decreasing and increasing emotional responses to unpleasant pictures. They found that the LPP amplitude was smaller under the decrease condition and larger under the increase condition relative to the passive view condition, with the effects beginning around 400 ms after picture onset and lasting for several seconds. However, recent studies (Paul, Simon, Kniesche, Kathmann, & Endrass, 2013; Schönfelder et al., 2014; Shafir et al., 2015; Thiruchselvam, Blechert, Sheppes, Rydstrom, & Gross, 2011) observed a smaller LPP elicited by negative pictures in the decrease reappraisal condition than in the viewing condition only during the late window of LPP (e.g., after 700 ms). The opposite LPP pattern has also been found. For example, Wu et al. (2013) used ERPs to investigate the time course of emotion regulation (decreasing, increasing, or maintaining negative emotion elicited by negative pictures) and found that the decrease condition evoked a larger LPP at 350–750 ms than the maintenance condition. Additionally, Wu et al. (2013) also found that the increase condition evoked more positive P2 (150–250 ms) and LPP (350–3,000 ms) than the maintenance condition.

This study employed ERP measures to examine how regulatory timing impacts the success of increase or decrease reappraisal. Similar to Urry (2009), we used unpleasant pictures as emotion‐eliciting stimuli. In contrast to Sheppes and Meiran (2007) and Urry (2009), this study presented emotion‐eliciting pictures for a shorter duration (4 s) and included more regulatory timing conditions. According to Urry (2009), this study took fully activation of emotion as the standard of division. In this study, early reappraisal introduction being before the high‐activation phase and late introduction being after the high‐activation phase. Based on the existing ERP research (Macnamara, Ochsner, & Hajcak, 2011; Moran, Jendrusina, & Moser, 2013; Moser et al., 2009; Schönfelder et al., 2014), the LPP peak of negative emotions appears in the 300–1,000 ms window after picture onset. So we speculated that the 300–1,000 ms interval might correspond to the high‐activation phase. Thus, in order to identify the best reappraisal timing for increasing or decreasing emotional responses to unpleasant pictures, in addition to anticipatory regulation (500 ms before picture onset), we set the timings of online regulation at three time points after the picture onset, which were 500 ms (in the high‐activation phase) and 1,500 ms and 2,000 ms (after the high‐activation phase). The anticipatory regulation (500 ms before picture onset) began before the high‐activation phase and so it was early introduction. Online 1,500 ms and 2,000 ms reappraisal began after the high‐activation phase and so they were late introduction. Online 500 ms reappraisal was in high‐activation phase. They formed four time points of reappraisal introduction. Specifically, Experiment 1 used two reappraisal initiation points (500 ms before picture onset and 2,000 ms after picture onset) and Experiment 2 used two other initiation points (500 ms and 1,500 ms after picture onset). Besides ERP measurement, this study also asked participants to rate their emotion after emotion regulation. Given the findings and viewpoints of Sheppes and Meiran (2007) and Urry (2009), we expected that decrease reappraisal would be more effective when initiated early rather than late, but increase reappraisal would be more effective when initiated in the high‐activation phase rather than early or late. Specifically, based on previous ERP studies (Bernat et al., 2011; Hajcak & Nieuwenhuis, 2006; Moser et al., 2009, 2014; Shafir et al., 2015), we expected that the LPP amplitude in central or parietal area would be more negative under the decrease instruction than increase or view instruction when initiated early, but would be more positive under increase instruction than decrease or view instruction when initiated in the high‐activation phase; in addition, the LPP amplitude in frontal area indexing cognitive effort would be larger under the increase or decrease instruction than view instruction.

## 2 MATERIALS AND METHODS

### 2.1 Participants

Sixteen participants (aged 18–25 years, mean age = 21 years) took part in Experiment 1. Another 18 participants (aged 19–25 years, mean age = 23 years) took part in Experiment 2. In order to rule out the potential influence of gender differences on emotional processing, only female participants were included in this study. Relative to males, it is easier to induce emotion in females, and females express emotion more easily (Sheppes et al., 2009). Previous studies have shown significant gender differences (Ray et al., 2010) in subjective rating of (Lang, Bradley, & Cuthbert, 2001) and physiological responses to (Bradley, Codispoti, Sabatinelli, & Lang, 2001) the materials from the International Affective Picture System (IAPS). Prior to the experiment, we referred to Keppel (1991) and determined our samples sizes (no less than 15, power > 0.80) in the participants through computation based on the results of pilot experiments. All participants in our study were right‐handed students from Capital Normal University in China and had normal or corrected‐to‐normal vision. The data from two participants were excluded from the final analysis because they did not meet our ERP analysis criterion of at least 16 trials per condition. Thus, Experiment 1 finally involved 15 participants and Experiment 2 involved 17 participants. All participants gave written informed consent for their participation in accordance with the Declaration of Helsinki. All methods were performed in accordance with the guidelines of human experimentation approved by the Institutional Review Board of Capital Normal University in China. No vulnerable populations were involved in this study. Each participant was paid 20 yuan per hour for their involvements after experiment.

### 2.2 Materials

Experiments 1 and 2 used the same stimuli, consisting of 80 negative pictures selected from the International Affective Picture System (IAPS) (Lang, Bradley, & Cuthbert, 1999) on the basis of IAPS normative data in China (Liu, Xu, & Zhou, 2009). According to the rerating of these pictures by Chinese university female students, the mean valence and arousal were 2.63 (*SD *= 0.45) and 5.32 (*SD *= 0.63), respectively. These negative pictures were divided into two groups, each group including 40 pictures, with no significant differences in valence (2.66 vs. 2.60) or arousal (5.32 vs. 5.33) between the two groups (*p*s > .10). The two groups were respectively used in two timing conditions. In Experiment 1, reappraisal instructions were delivered at 500 ms before (i.e., anticipatory condition) or 2,000 ms after (i.e., online 2,000 ms condition) picture onset. In Experiment 2, reappraisal instructions were delivered at 500 ms (i.e., online 500 ms condition) or 1,500 ms (i.e., online 1,500 ms condition) after picture onset. In order to avoid systematic variation, picture groups were counterbalanced to appear in both timing conditions of each experiment. Forty neutral pictures (valence *M* = 5.12, arousal *M* = 4.34) were selected from IAPS as filler stimuli. Practice materials consisted of another 20 negative and 10 neutral pictures from IAPS.

In each experiment, each negative picture was presented three times: once under the condition of increase reappraisal (increase‐negative trials), once under decrease reappraisal (decrease‐negative trials), and once in the viewing condition (view‐negative trials). Each neutral picture was presented twice, once in each timing condition (all were view‐neutral trials). Each participant was required to complete 320 trials—80 increase‐negative trials, 80 decrease‐negative trials, 80 view‐negative trials, and 80 view‐neutral trials. In Experiment 1, half of the trials were in the anticipatory condition and the other half were in the online 2,000 ms condition. In Experiment 2, half of trials were in the online 500 ms condition and the other half were in the online 1,500 ms condition. The trials were divided into eight blocks, with 40 trials in each block. The order of trials was pseudorandom, and no more than two trials with the same timing occurred successively in the trial sequences. Additionally, we separated the increase and decrease trials by view‐neutral and view‐negative trials to avoid the difficulties brought about by direct task switching (i.e., switching from increasing to decreasing or from decreasing to increasing on successive trials).

### 2.3 Procedures

Before the formal experiment, the participants were trained to follow one of three instructions during each trial: increase, decrease, or view. On the increase trials, after participants saw the reappraisal cue (upward arrows “↑↑”), they were instructed to imagine that they were in the situation depicted by the negative pictures, even being the central figure in a picture, or to imagine the depicted situation getting worse and people or animals in the pictures being in great pain or danger. On decrease trials, after the reappraisal cue (downward arrows “↓↓”) was presented, the participants were instructed to view pictured events from a detached, third‐person perspective and to imagine that the pictured events were fake or made up, or to imagine the depicted situation getting better (for example, the people in the pictures would soon get relief). On view trials, after the cue (horizontal lines “=”) was presented, the participants were instructed to passively look at negative or neutral pictures without trying to change the way they thought about them. All participants reported that they could use the suggested reappraisal strategies after the practice trials were completed.

**Figure 1:**
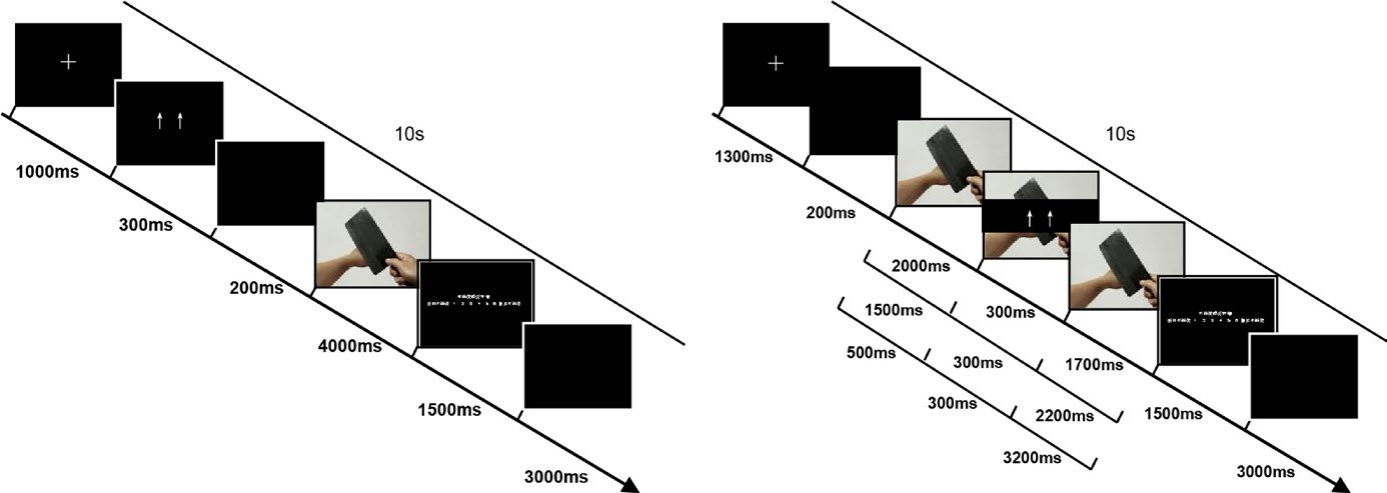
Schematic representations of a trial in the anticipatory condition (the left) and online 500/1,500/2,000 ms condition (the right). See text for details. The example picture resembles those in the experiment but it is not part of the IAPS

Under the anticipatory condition (see the left in Figure 1), a trial began with a white fixation point presented for 1,000 ms. Following the fixation, a regulation cue (↑↑, =, or ↓↓) was displayed on the screen for 300 ms, and then it was replaced by blank screen for 200 ms. Next, a picture was presented for 4,000 ms and participants were asked to regulate their emotions according to the regulation cue. After the picture disappeared, an emotion‐rating scale ranging from 1 (“not unpleasant”) to 6 (“extremely unpleasant”) was displayed on the screen for 1,500 ms. Participants had to press one of six keys to indicate their emotion. The next trial began after a blank screen for 3,000 ms. Under the online 500 ms, 1,500 ms, and 2,000 ms conditions (Figure 1, right), each trial began with a fixation point presented for 1,300 ms, followed by a blank screen for 200 ms and then by a picture for 500 ms, 1,500 ms, or 2,000 ms. Next, a blank rectangle with a regulation cue was superimposed onto the central part of the picture. After 300 ms, the cue disappeared, while the picture remained on the screen for an additional 3,200 ms, 2,200 ms, or 1,700 ms. Participants were instructed to regulate their emotions according to the regulation cue. Then, the 6‐point emotion‐rating scale was presented for 1,500 ms, and participants had to press one of the six keys to indicate their emotion; this was followed by a blank screen for 3,000 ms.

At the end of each block, a six‐point scale (1 = “little effort,” 6 = “most effort”) was used to rate participants’ effort level. After all eight blocks were completed, the participants were required to look at 10 pictures that had been presented in the previous blocks and report aloud the specific strategy they had used for each picture.

### 2.4 ERP recordings and analysis

Electroencephalograms were recorded by an amplifier (Neuroscan SynAmps2; NeuroScan, Inc., Sterling, Virginia, USA) from 64 Ag/AgCl electrodes positioned in an electrode cap according to the international 10–20 system. All scalp electrodes were referenced online to the left mastoid and rereferenced offline to the average of the right and left mastoid recordings. Vertical and horizontal electrooculograms were recorded with two pairs of electrodes, one pair placed above and below the left eye and another pair at the outer canthi of both eyes. EEG signals were sampled at a rate of 500 Hz and filtered with a bandpass of 0.01–40 Hz. Impedances were kept below 5 kΩ. The preprocessing of the ERP data was conducted using Scan 4.5 software. Electrooculograph blink artifacts were corrected using a linear regression estimate. Trials with voltages exceeding ±75 μV were excluded from ERP analysis as artifacts. Each averaging epoch lasted 4,200 ms, with 200 ms prior to picture onset serving as a baseline.

In Experiment 1 and Experiment 2, according to the previous studies (Cuthbert et al., 2000; Moser et al., 2009), the central‐parietal and parietal LPP generally was associated with emotional intensity, therefore, we selected six electrodes (C3, Cz, C4, P3, Pz, and P4) from the left hemisphere, middle, and right hemisphere at central and parietal locations; meanwhile, the related studies suggested enhanced frontal LPP may index increased cognitive effort associated with reappraisal (Bernat et al., 2011; Moser et al., 2014; Shafir et al., 2015), so we also chose three frontal electrodes (F3, Fz, and F4) for the LPP. In addition, based on careful examination, we also selected other three electrodes (FP1, FPz, and FP2) at the prefrontal location in Experiment 1. Average amplitudes over left, midline, and right electrodes in each location were calculated with reference to a 200 ms prepicture baseline, with time windows based on visual inspection of the timing of waveforms. Repeated‐measures analyses of variance (ANOVAs) were conducted in each time window. The alpha level was .05. The *p* values were corrected using the Greenhouse–Geisser method. All significant main effects of goal were supplemented with multiple comparisons using Bonferroni correction. Significant or marginally significant interactions between goal and location were supplemented with simple effect analyses also using Bonferroni correction. We did not report on the main effects of location.

According to Simmons, Nelson, and Simonsohn (2012), we explicitly stated that we reported how we determined our sample size, all data exclusions, all manipulations, and all measures in the study.

## 3 RESULTS

### 3.1 Behavioral data

#### 3.1.1 Experiment 1

The average emotion‐rating scores under the different conditions in Experiment 1 are shown in Table 1. The 2 (regulatory timing: anticipatory, online 2,000 ms) × 3 (regulatory goal: increase, decrease, view) repeated‐measures ANOVA on emotion‐rating scores revealed a significant main effect of goal [*F*_2,28_ = 110.91, *p* < .001, η_*p*_^2^ = 0.89]. Multiple comparisons showed that participants’ emotion reported after the increase reappraisal was more negative than that after the decrease reappraisal and under the view condition (*p*s < .001), and participants’ emotion reported under the view condition was more negative than that after the decrease reappraisal (*p* < .001). However, main effect of timing and interaction between timing and goal did not reach significance [*F*_1,14_ = 0.24, *p* = .629, η_*p*_^2^ = 0.02; *F*_2,28_ = 1.37, *p* = .271, η_*p*_^2^ = 0.09].

**Table 1:** Participants’ emotion‐rating scores after reappraisal in Experiments 1 and 2

Goal	Experiment 1		Experiment 2	
	Anticipatory	Online 2,000 ms	Online 500 ms	Online 1,500 ms
Increase	5.14 ± 0.17	5.12 ± 0.17	4.74 ± 0.17	4.86 ± 0.17
View	3.19 ± 0.18	3.14 ± 0.20	2.75 ± 0.24	2.74 ± 0.23
Decrease	1.79 ± 0.10	1.83 ± 0.11	2.25 ± 0.20	2.21 ± 0.20

The data in the table are the standard errors.

#### 3.1.2 Experiment 2

The average emotion‐rating scores under the different conditions in Experiment 2 also are shown in Table 1. The 2(regulatory timing: online 500 ms, online 1,500 ms) × 3 (regulatory goal: increase, decrease, view) repeated‐measures ANOVA on emotion‐rating scores revealed significant main effect of goal [*F*_2,32_ = 55.914, *p* < .001, η_*p*_^2^ = 0.778] and interaction between timing and goal[*F*_2,32_ = 6.249, *p* = .008, η_*p*_^2^ = 0.281]. Further analysis showed that participants’ emotion reported after the increase reappraisal was more negative than that after the decrease reappraisal and under the view condition (*p*s < .001), regardless of timing condition. However, differences between decrease and view under two timing conditions did not reach significance (*p* = .307; *p* = .232). Additionally, when regulatory goal was to increase negative emotion, participants reported more negative emotion under the online 1,500 ms condition than under the online 500 ms condition. Actually, this difference was very small (4.86 vs. 4.74) although significant (*p* = .004).

In addition, participants reported that, when regulating their emotions according to the instruction, their effort levels in Experiments 1 and 2 (*M* = 5.09 and *M* = 5.12) both were very high. The check of emotion regulation strategies used by the participants showed that all participants had carried out emotion regulation in accordance with the experimental instructions.

### 3.2 ERP result

#### 3.2.1 Experiment 1

The 3 (regulatory goal: increase, decrease, view) × 4 (location: prefrontal, frontal, central, parietal) repeated‐measures ANOVAs were conducted in every time window.

##### ERP data under the anticipatory condition

**Figure 2:**
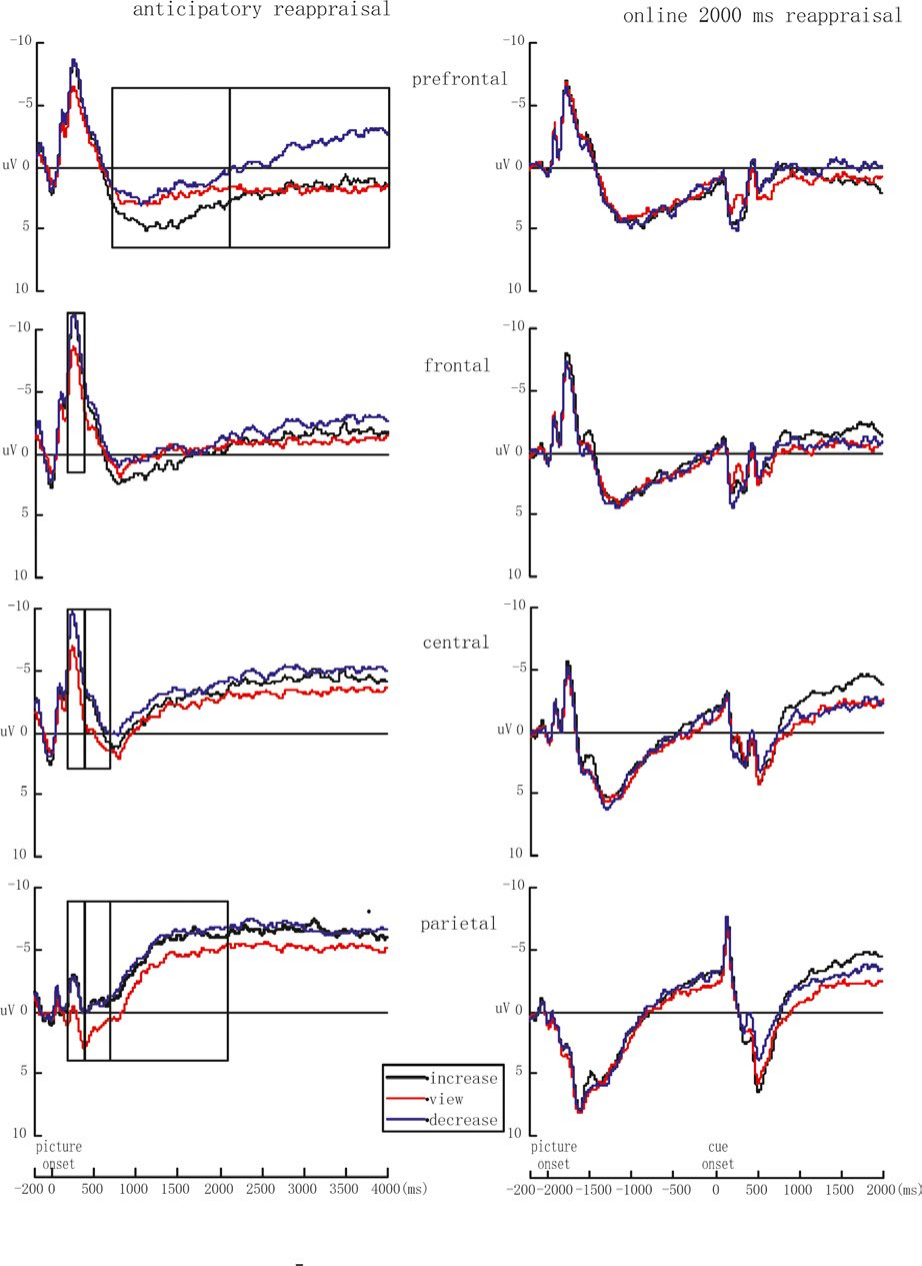
ERP comparison of three regulatory goals under the anticipatory condition (the left) and online 2,000 ms condition (the right). Under anticipatory reappraisal, the more negative ERPs elicited by the increase and decrease reappraisal than view condition were found during 200–400 ms postpicture in the frontal, central, and parietal areas, and the pattern remained obvious in the parietal area during 400–700 ms; the increase reappraisal yielded a more positive prefrontal LPP than decrease reappraisal, and relative to view condition, decrease reappraisal evoked a more negative parietal LPP during 700–2,100 ms; a more negative LPP evoked by decrease reappraisal than the increase reappraisal and view condition was showed during 2,100–4,000 ms, only in prefrontal area. Under the online 2,000 ms condition, no significant effects of goal were showed at every time window

Based on a careful examination of our grand average waveforms (see Figure 2), this study evaluated the time windows of 200–400 ms, 400–700 ms, and LPP under the anticipatory reappraisal. The average waveforms during 200–400 ms appear to be capturing N2 component. The LPP is frequently analyzed in various time windows (Langeslag & Van Strien, 2010; Moser et al., 2009). The LPP amplitude of this study was computed at two different time windows (700–2,100 ms and 2,100–4,000 ms postpicture).

During 200–400 ms, our analysis indicated that the interaction of goals and location was marginally significant [*F*_6,84_ = 2.47, *p* = .092, η_*p*_^2^ = 0.15]. Simple effect analysis showed significant effects of goals in frontal area, central area, and parietal area, with more positive ERP under the view condition than under the increase and decrease conditions (*p* = .002, *p* = .008; *p* = .001, *p* = .002; *p* < .001, *p* = .002).

During 400–700 ms, our analysis revealed a significant interaction between goal and location [*F*_6,84_ = 4.34, *p* = .009, η_*p*_^2^ = 0.24]. Further analysis showed that the ERP under the view condition was more positive than that under the decrease condition in central area (*p* = .020). In parietal area, we observed more positive ERP under the view condition than under decrease and increase conditions (*p* *=* .014*; p* *=* .018).

During 700–2,100 ms, our analysis showed a significant interaction between goals and location [*F*_6,84_ = 5.15, *p* = .007, η_*p*_^2^ = 0.27]. Further analysis indicated that the ERP amplitude under the increase condition was more positive than that under the decrease condition in prefrontal area (*p* = .040) and the ERP under the view condition was more positive than that under the decrease condition in parietal area (*p* = .039).

During 2,100–4,000 ms, our analysis showed a marginally significant interaction between goal and location [*F*_6,84_ = 2.85, *p* = .069, η_*p*_^2^ = 0.17]. Further analysis revealed that, only in prefrontal area, the average amplitudes under the increase and view conditions were more positive than that under the decrease condition (*p* = .013; *p* = .007).

##### ERP data under the online 2,000 ms condition

On the basis of visual inspection of the grand average waveforms, mean amplitudes were computed at three time windows (450–750 ms, 750–1,100 ms, and 1,100–2,000 ms after regulatory cue onset) under online 2,000 ms condition. Our results showed that no significant main effect of goal and interaction between goal and location (*p*s > .05), as shown in Figure 2.

#### 3.2.2 Experiment 2

Three (regulatory goal: increase, decrease, view) × 3 (location: frontal, central, parietal) repeated‐measures ANOVAs were conducted in every time window.

##### ERP data under the online 500 ms condition

**Figure 3:**
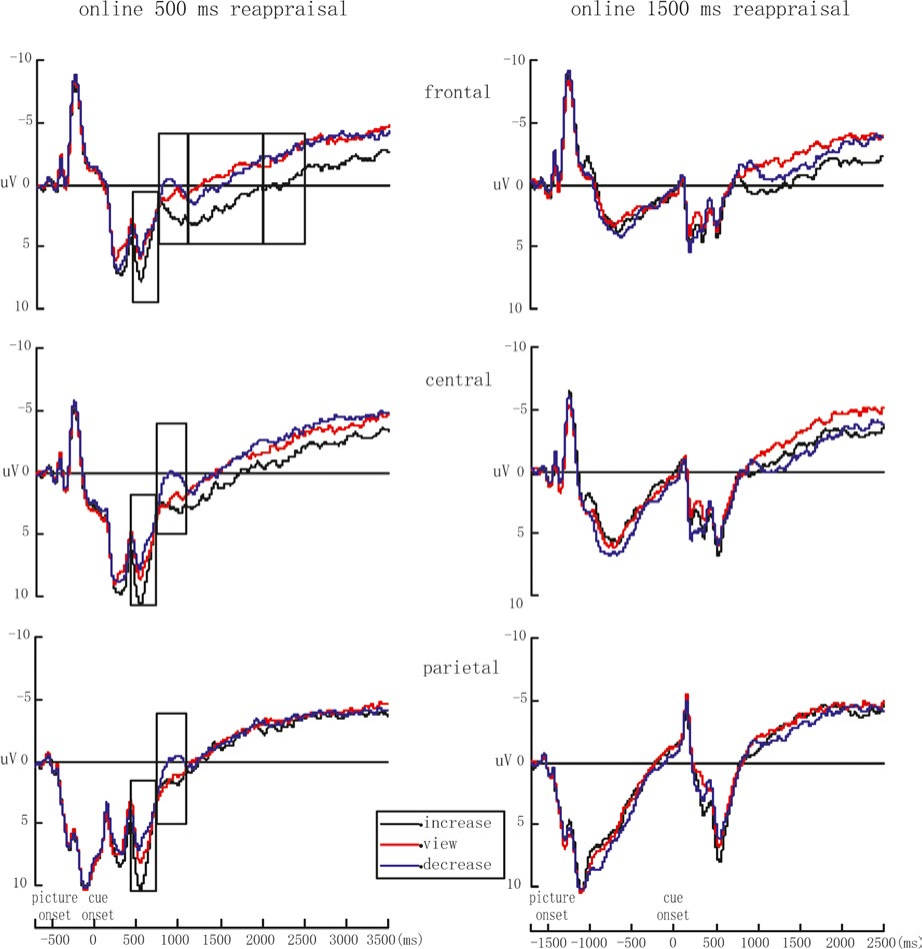
ERP comparison of three regulatory goals under the online 500 ms condition (the left) and online 1,500 ms condition (the right). Under the online 500 ms condition, the frontal LPP under increase reappraisal was more positive than under the decrease or view conditions from 450 ms to 2,500 ms after regulatory cue onset; the increase reappraisal evoked a larger centro‐parietal LPP than the decrease reappraisal and view conditions during 450–750 ms, and during 750–1,100 ms, the increase reappraisal continued to evoke a lager centro‐parietal LPP than the decrease reappraisal. Under the online 1,500 ms condition, no significant effects of goal were found

Based on a careful examination of our grand average waveforms (see Figure 3), the LPP amplitude was computed at six different time windows (450–750 ms, 750–1,100 ms, 1,100–2,000 ms, 2,000–2,500 ms, 2,500–3,000 ms, and 3,000–3,500 ms post regulatory cue onset).

During 450–750 ms, our analysis indicated a significant main effect of goal [*F*_2,32_ = 5.46, *p* = .012, η_*p*_^2^ = 0.25] and not significant interaction between goal and location[*F*_4,64_ = 1.44, *p* = .248, η_*p*_^2^ = 0.08]. Further analysis in main effect of goal showed that, in frontal, central, parietal area, the average amplitude under the increase condition was significant more positive than those under the view and decrease conditions (*p* = .050; *p* = .018).

In the 750–1,100 ms time window, our analysis indicated a significant main effect of goal [*F*_2,32_ = 4.26, *p* = .033, η_*p*_^2^ = 0.21] and a marginally significant interaction between goal and location [*F*_4,64_ = 2.63, *p* = .076, η_*p*_^2^ = 0.14]. Further analysis showed that the ERP amplitudes under the increase condition were more positive than that under the decrease condition in frontal area, central area, and parietal area (*p* = .035; *p* = .013; *p* = .008).

During 1,100–2,000 ms, our analysis revealed a significant interaction between goal and location [*F*_4,64_ = 5.98, *p* = .002, η_*p*_^2^ = 0.27]. Further analysis showed that the average amplitude under the increase condition was more positive than those under the view and decrease conditions only in frontal area (*p* = .008; *p* = .042).

During 2,000–2,500 ms, our analysis showed a significant interaction between goal and location [*F*_4,64_ = 3.91, *p* = .020, η_*p*_^2^ = 0.20]. Further analysis revealed that the average amplitude under the increase condition was significant more positive than those under decrease conditions only in frontal area (*p* = .013).

During 2,500–3,000 ms and 3,000–3,500 ms, our analysis showed respectively significant interactions between goal and location [*F*_4,64_ = 5.54, *p* = .002, η_*p*_^2^ = 0.26; *F*_4,64_ = 2.93, *p* = .058, η_*p*_^2^ = 0.16]. Further analysis revealed that there were no differences between the three regulation goals in frontal, central, and parietal areas (*p*s > .05).

##### ERP data under the online 1,500 ms condition

Mean amplitudes were computed at four time windows (450–750 ms, 750–1,100 ms, 1,100–2,000 ms, and 2,000–2,500 ms after regulatory cue onset) for each subject and condition. The ANOVAs in these time windows showed no significant main effect of goal and interaction between goal and location (*p*s > .05), as shown in Figure 3.

The main results of ERP comparison between three regulatory goals under different regulatory timings condition in Experiments 1 and 2 were listed in Table 2. Because no significant differences between three regulatory goals were found in 2,500–3,000 ms and 3,000–3,500 ms under the online 500 ms condition and in all the time windows under the online 1500 ms and online 2,000 ms condition, the results under these conditions were not listed in the table.

**Table 2:** ERP comparison of three regulatory goals respectively under the anticipatory condition and the online 500 ms condition

Anticipatory					Online 500 ms			
Time window	Prefrontal	Frontal	Central	Parietal	Time window	Frontal	Central	Parietal
200–400 ms		V > I V > D	V > I V > D	V > I V > D	450–750 ms	I > V I > D	I > V I > D	I > V I > D
400–700 ms			V > D	V > I V > D	750–1,100 ms	I > D	I > D	I > D
700–2,100 ms	I > D			V > D	1,100–2,000 ms	I > V I > D		
2,100–4,000 ms	I > D V > D				2,000–2,500 ms	I > D		

“V,” “I,” and “D” respectively suggested regulatory goal of “view,” “increase,” and “decrease.” “>” indicated “significantly more positive,” *p*s < .05.

## 4 DISCUSSION

The present study employed ERP measures to test the hypothesis that decrease reappraisal would be more effective when initiated early relative to late, but increase reappraisal would be more effective when initiated in the high‐activation phase. The most important finding of this study was that more positive ERP was associated with increase instructions than with decrease and view instructions mainly under the online 500 ms reappraisal condition, and more negative ERP was associated with decrease instructions than with increase and view instructions mainly under the anticipatory reappraisal condition. These results partially support our hypothesis. The present study also collected self‐reported negative experience after emotion regulation. The following is a detailed discussion of our results.

The results of Experiment 1 showed that participants’ negative experience was stronger under the increase condition and weaker under the decrease condition relative to the view condition, regardless of the timing. However, Experiment 2 showed that under the online 500 ms or 1,500 ms reappraisal condition, the enhancement effect of increase reappraisal was more obvious, but no reduction effect of decrease reappraisal was found. The behavioral results of the two experiments were inconsistent, which was related to the timings when reappraisal was introduced. According to the microscopic cycle model of nonlinear emotional activation (Urry, 2009), emotional activation increases from low‐level to a high‐level peak for some time, then returns to the initial low level. In the present study, at 500–1,500 ms after picture onset, emotion may be fully activated, making increase reappraisal easy to achieve, but making decrease reappraisal exceptionally difficult. On the other hand, when online 2,000 ms reappraisal was introduced, the level of negative emotional activation may have declined somewhat, but not to the initial low level; therefore, subjective emotions were enhanced by increase reappraisal or reduced by decrease reappraisal to some degree. The behavioral results also showed that under the increase condition, the ratings of online 1,500 ms reappraisal were higher than those of online 500 ms reappraisal, but the difference between the two was only 0.113, possibly due to the bias of feelings and self‐report (Sheppes & Meiran, 2007). However, the above behavioral results were partially supported by our ERP results. We are much more interested in ERPs than in self‐reported emotional intensity because self‐reports of emotional experience may not be sensitive enough (Urry, 2009).

The ERP results of the anticipatory reappraisal showed that in the frontal, central, and parietal areas, increase and decrease reappraisals elicited a larger negative component than the view condition in N2 component during 200–400 ms postpicture, and the pattern remained obvious in the parietal area during 400–700 ms. The larger negative trend of N2 was considered to reflect recruitment of greater attentional resource (Scudder, Drollette, Pontifex, & Hillman, 2012; Zhang, Kang, Wu, Ma, & Guo, 2015). Reappraisal required participants to reinterpret emotion‐inducing events; therefore, in the beginning period of picture presentation, participants had to identify the picture and change the meaning of the picture. The association of both increase and decrease reappraisals with more negative ERPs may reflect the mental operations of identifying and then changing the meaning of the picture under reappraisal.

Similar to previous studies (e.g., Moser et al., 2006, 2009; Schönfelder et al., 2014), the present study found a more negative parietal LPP during 700–2,100 ms under the decrease condition than under the view condition, suggesting that reappraisal successfully decreased negative emotion under anticipatory regulation. In the same time window, the decrease condition evoked a smaller prefrontal LPP than the increase condition. According to related recent studies (Bernat et al., 2011; Moser et al., 2014; Shafir et al., 2015), enhanced frontal LPP may indicate increased cognitive effort associated with reappraisal. Employing this explanation, the larger prefrontal LPP for increase reappraisal than for decrease reappraisal in the present study suggests that increase reappraisal consumed more cognitive resources, although it did not effectively enhance negative emotions.

In the subsequent time window (i.e., 2,100–4,000 ms), our ERP results for the anticipatory reappraisal showed that the decrease condition evoked a smaller prefrontal LPP than the increase and view conditions. No significant difference in prefrontal LPP was observed between increase and view conditions. We suspected that this late prefrontal LPP amplitude reflected the degree of mental conflict. The decrease reappraisal conforms to the daily needs of emotional regulation and can successfully diminish participants’ negative emotion, so it may make participants experience less mental conflict. However, increasing negative emotion goes against the instincts of self‐preservation, causing resistance and more mental conflict in participants. As a result, prefrontal LPP was more positive under the increase condition than under the decrease condition. In addition, compared with decrease reappraisal, participants under the passive viewing condition also suffered more conflict because they did not actively regulate their emotions.

The ERP results of the online 500 ms reappraisal showed that increase reappraisal evoked a larger centro‐parietal LPP than the decrease reappraisal and view conditions during 450–750 ms after regulatory cue onset, and during 750–1,100 ms, increase reappraisal continued to evoke a lager centro‐parietal LPP than decrease reappraisal. These results suggest that reappraisal successfully increased negative emotion under the online 500 ms condition. Meanwhile, the frontal LPP was more positive under increase reappraisal than under the decrease or view conditions for a period of several seconds beginning at 450 ms after regulatory cue onset, possibly reflecting increased cognitive effort and more mental conflict associated with increase reappraisal.

Combining the results of Experiments 1 and 2, the present study showed that when one intends to reduce emotional response, anticipatory reappraisal may be more effective than online reappraisal, whereas when one intends to increase emotional response, online 500 ms reappraisal may be most effective. These findings support the idea that regulatory timings have an important influence on the effect of cognitive reappraisal and the impact of regulatory timing depends on one's regulatory goal. Unlike previous studies which only showed that the effect of decrease reappraisal is moderated by regulatory timing (Sheppes & Meiran, 2007) or that the effectiveness of increase reappraisal depends on regulatory timing (Urry, 2009), the present study revealed that the effects of both decrease and increase reappraisals are moderated by regulatory timing. Consistent with Sheppes and Meiran (2007) and our expectations, the present study showed that decrease reappraisal is effective in reducing negative experience when initiated in advance, which may be because the emotion is not activated at that point and this state is easy to reach decrease regulatory goal, based on Urry's (2009). However, Sheppes and Meiran (2007) found that decrease reappraisal was also effective when the reappraisal instruction was delivered to participants 37.5 s after film onset. The different results of Sheppes and Meiran (2007) and the present study could be due to the use of different emotion‐inducing stimuli.

In Urry's study (2009), reappraisal was introduced 2 s before or 4 s after negative picture onset. Urry (2009) found that compared to the maintenance condition, higher corrugator muscle activity and electrodermal activity elicited by increase reappraisals were only apparent under the online timing condition. Similar to that study, the present study also showed that online increase reappraisal was effective. However, while Urry (2009) used only one online timing condition, the present study set three online timing conditions and found that only the online 500 ms reappraisal was effective in enhancing negative emotion. One possible explanation, based on Urry's (2009) microscopic cycle model of nonlinear emotional activation and Gross's (1998b) process model of emotion regulation, is that when the reappraisal instruction was delivered to participants 500 ms after regulatory cue onset, the emotional activation was increasing, which was consistent with the regulatory goal, so the increase reappraisal was easy to implement. However, when the reappraisal instruction was delivered to participants 1,500 ms or 2,000 ms after regulatory cue onset, the emotional activation had reached its highest level or begun to drop; at this point, increase reappraisal was difficult to implement. These results suggest that online 500 ms after picture onset may be the “point of no return” (Sheppes & Meiran, 2007), at which time the emotion is fully activated and decrease reappraisal therefore becomes exceptionally difficult.

When should reappraisal be introduced for the best effect? Answering this question has been a focus of both theorists and clinicians. The previous studies used the subjective emotion experience and physiology (corrugator activity, heart rate, and electrodermal activity) as the indicators to examine the influence of regulatory timing on effectiveness of reappraisal. However, our study using ERP found that regulatory timing has an important influence on the effect of cognitive reappraisal and anticipatory reappraisal has an advantage in reducing negative emotion, and online 500 ms reappraisal facilitates increasing negative emotion. Decrease reappraisal is most effective when induced in advance or early at an unactivated stage of negative emotion. Online (500 ms) increase reappraisal is most effective once the negative emotion has been activated and becomes ineffective afterward. There seems to exist a “point of no return” at which emotion regulation efforts are challenged and which occurs at the highest level of emotional activation. Decrease reappraisal is more effective before this point, but increase reappraisal is more effective close to this point.

## 5 CONCLUSION

The present study used ERPs to determine the best timing of reappraisals. Our ERP results showed that, relative to view condition, decrease reappraisal evoked a more negative parietal LPP during 700–2100 ms following picture onset under the anticipatory regulation, and increase reappraisal evoked a more positive centro‐parietal LPP during 450–750 ms after regulatory cue onset under the online 500 ms regulation. These results suggest that reappraisal successfully decreased negative emotion under the anticipatory regulation and successfully increased negative emotion under the online 500 ms regulation. Moreover, the present study showed that increase reappraisal evoked a larger LPP than decrease reappraisal in prefrontal area beginning at about 700 ms after picture onset under the anticipatory regulation condition and in frontal area beginning at 450 ms after regulatory cue onset under the online 500 ms regulation condition might reflecting increased cognitive effort and more mental conflict associated with increase reappraisal. However, one limitation of the present study is that ERP results are inconsistent with behavioral results. Future studies are needed to help clarify the inconsistency. Another limitation of the present study is the lack of male participants. Therefore, our findings are limited to females.

## CONFLICT OF INTEREST

The authors declare no competing financial interests.

## AUTHOR CONTRIBUTIONS

Q.Z. supervised the project. C.Y. analyzed the data and wrote the main manuscript text and prepared Figures 1, 2, 3 and Tables 1 and 2. N.L. collected the data. Q.Z. and L.C. revised the draft of manuscript. Besides, all authors reviewed the manuscript.
